# A comment on 'Theegarten et al.: Submesothelial deposition of carbon nanoparticles after toner exposition: Case report. Diagnostic Pathology 2010, 5:77'

**DOI:** 10.1186/1746-1596-6-20

**Published:** 2011-03-17

**Authors:** Michael Wensing, Tobias Schripp, Erik Uhde, Tunga Salthammer

**Affiliations:** 1 Department of Material Analysis and Indoor Chemistry, Fraunhofer WKI, Bienroder Weg 54E, 38108 Braunschweig, Germany; 2 International Laboratory for Air Quality and Health, Queensland University of Technology (QUT), GPO Box 2434, Brisbane 4001, Australia

## 

A comment on 'Theegarten et al.: Submesothelial deposition of carbon nanoparticles after toner exposition: Case report. Diagnostic Pathology 2010, 5:77'

We read with interest the article by Theegarten et al. [1] which reports on the case of a patient in whose peritoneum were found submesothial aggregates of carbon nanoparticles (CNPs) with a diameter of 31-67 nm. From their literature study and results the authors conclude that these CNPs must be particles emitted by a laser printer in an office environment and inhaled by the patient. In addition the abstract for [1] states: *"Inhalation of CNP from toner dust has been shown to have impact on the respiratory health of persons exposed"*. Other possible exposure and uptake paths for CNPs are not discussed in [1].

The paper by Theegarten et al. [1] includes experimental data on the morphology and on the constituents of the toner powder of a laser printer. This toner consists of particles with a diameter of 5-9 μm on whose surface "*some small elevations" *of metal oxides are found. The CNPs detected in the patient's peritoneum were not, however, identified as toner constituents.

In fact the assumption by Theegarten et al. that the CNPs detected in the patient's peritoneum come from a laser printer is based solely on a reference to a study by Wensing et al. [2]. Theegarten et al. refer to [2] as follows: *"Office printers were detected to emit carbon nanoparticles (CNP) in a variable extend"*. As the authors of [2] we should like to note at this point that this statement is not correct. Our publication [2] does not contain any data which would point to CNPs being possible constituents of ultra-fine particles released from laser printers.

Our chamber experiments were carried out in a clean and particle-free atmosphere. Therefore we can exclude influences from other sources and outdoor air. Indeed our article [2] states that the ultra-fine particles (UFPs) emitted from laser printers are volatile and are *not *original toner constituents ("toner dust"). The UFPs are rather formed by the nucleation and condensation processes of hydrophobic SVOCs (semi-volatile organic compounds) such as, for example, semi-volatile organic silicon compounds and higher aliphatic hydrocarbons [3,4]. The volatility of the UFPs - which is also inconsistent with CNP as constituents of the UFPs - was confirmed by VH-TDMA (volatilization and humidification tandem differential mobility analyzer) [4] and thermo denuder [3] measurements. We have never found CNPs in connection with laser printer experiments. Paper [4] is also cited in Theegarten et al. [1].

To sum up, it can be stated that our experimental data published in [2] and also in our later publications [3] and [4] do not in any way support the proposition that the patient in the present case was exposed to CNPs from laser printers.

## Competing interests

The authors have been working for many years at the Fraunhofer WKI and among other things are also involved in research projects financed by industry. A project concerning particle emissions from laser printers is currently on the point of completion. This project has been commissioned and financed by thirteen laser printing and copying equipment manufacturers organized within BITKOM, the Federal Association for Information Technology, Telecommunications and New Media. We will shortly be publishing the results of this project. However, the results reported in [2] are not part of the BITKOM project.
